# Burnout in medical undergraduate students in Qassim, Saudi Arabia

**DOI:** 10.1186/s43045-021-00128-2

**Published:** 2021-09-06

**Authors:** Abdullah Alqifari, Mashael Alghidani, Ruba Almazyad, Aljowharah Alotaibi, Wijdan A. Alharbi, Entisar Aljumail, Ghaida Alqefari, Abdulmajed Alkamees, Hana Alqifari

**Affiliations:** 1 Psychiatry Department, College of Medicine, Qassim University, Buraydah, Qassim Saudi Arabia; 2 Internal Medicine Department, Qassim University, Ministry of Health, Buraydah, Saudi Arabia; 3 College of Medicine, Qassim University, Buraydah, Qassim Saudi Arabia; 4 King Abdul-Aziz Medical City, Ministry of National Guard – Health Affairs, Riyadh, Saudi Arabia; 5 Family Medicine Academy, Ministry of Health, Buraydah, Qassim Saudi Arabia; 6 Qassim University, Buraydah, Qassim Saudi Arabia; 7 Psychiatry Department, College of Medicine, Qassim University, Buraydah, Qassim Saudi Arabia; 8 College of Science, Department of Statistics, Qassim University, Buraydah, Qassim Saudi Arabia

**Keywords:** Burnout, Medical students, Depersonalization, Medical education

## Abstract

**Background:**

Burnout, defined as mental and physical exhaustion, has been an issue for many medical students. Medical student burnout is associated with many factors such as academic pressure, sleep deprivation, exposure to patient suffering, and high academic demand. In this study, we assessed the prevalence of burnout symptoms among preclinical and clinical medical students studying at Qassim University in Qassim, Saudi Arabia.

**Results:**

Three hundred thirty-six subjects entered the final data analysis with a majority between 18 and 24 years of age, of whom 56.5% was females and 43.5% was males. The overall burnout prevalence was 8%. The female gender was a significant predictor of emotional exhaustion and personal efficacy, (OR = 2.510; 95% Cl [1.845–3.415]; *p* value 0.000) and (OR = 1.434; 95% Cl [1.086–1.866]; *p* value 0.010), respectively.

**Conclusion:**

Among medical students, burnout is common. The impact of gender on burnout was noticed; female gender was a significant predictor of emotional exhaustion and personal efficacy. Medical education style had no impact on burnout levels among medical students.

## Background

Medical colleges train medical students to take care of patients, work on research, and educate the public about health-related issues [1]. The demand for medical students to achieve these goals is high, and they are at risk of burnout as a result [1]. Burnout is included in the 11th Revision of the International Classification of Diseases (ICD-11) as an occupational phenomenon rather than a medical condition. It is described as a syndrome conceptualized as a consequence of chronic stress in the workplace that is not effectively handled. The affected person’s energy, attitudes, perceptions, or function are adversely influenced. Burnout has three dimensions: depletion or exhaustion of energy, increasing mental distance from work or negative or cyclical feelings related to work, and decreased professional efficacy [2]. The gold standard instrument for burnout is the Maslach Burnout Inventory (MBI) [3]. The MBI has three dimensions: emotional exhaustion (EE), depersonalization, and a decreased sense of accomplishment [3]. Some studies have shown that student psychological distress is correlated with decreased academic success, increased academic dishonesty, elevated drug misuse, cynicism, unwillingness to care for the chronically ill, diminished empathy, and suicidal ideation [1 4]. Burnout is prevalent among medical students. A systemic review that included nine studies reported a prevalence range between 45 and 71% [4].

Problem-based learning (PBL) is a teaching approach in medical schools where real-world clinical issues facilitate students’ learning instead of the straightforward introduction of facts and concepts of conventional teaching. Studies have been conducted to determine whether medical curricula modifications minimize students’ psychological distress and increase productivity, but the findings remain collectively inconclusive [5].

A more modern form of education is team-based learning (TBL). TBL is student-centered but guided by a teacher. TBL promotes person and group responsibility as small groups of students work together to address questions [6].

In Saudi Arabia, Qassim University has two medical colleges. Al-Mulayda Medical College uses the PBL teaching method, and Unayzah Medical College uses the TBL style.

To our knowledge, no previously published research has studied the differences in Qassim medical students’ burnout between its two medical colleges. This study aimed to assess the prevalence of burnout among Qassim medical students and its associated factors, including the impact of medical education used.

## Methods

### Study design and setting

We conducted a cross-sectional study between 2-3-2020 and 23-3-2020 at the College of Medicine at Qassim University in the Qassim region, Saudi Arabia. Qassim University has two medical colleges. The leading medical college, located at Al-Mulayda, began its 5-year Bachelor of Medicine and Surgery (MBBS) program in the academic year 2000–2001. It is the first college in Saudi Arabia to adopt problem-based learning (PBL) and implement an integrated curriculum. Unayzah Medical College was established in 2011. It is the first college in Saudi Arabia to adopt the team-based learning (TBL) style as an essential foundation for medical curricula.

### Participants, sampling method, and sample size

Undergraduate students at the Medical College of Qassim University were invited to participate in a Web-based survey. We provided an incentive (iPad) for one of the study participants selected randomly at the end of the sample collection to encourage participation. A total of 409 participants responded to the questionnaire out of 393 medical students in Unayzah Medical College and 726 medical students in Al-Mulayda Medical College. Students who neither complete all items of the MBI-SS or the demographic information were excluded, leaving a total of 336 participants to include in the analysis: first-year (*n* = 42), second-year (*n* = 91), third-year (*n* = 61), fourth-year (*n* = 68), fifth-year (*n* = 56), and internship (*n* = 18) undergraduate medical students. The sample size of 287 medical students from Buraidah and Unayzah Medical College was determined using Cochran’s formula with a 5% margin of error and a 95% confidence level.

### Ethical considerations

Ethical approval was obtained from the Institutional Review Board at Qassim University. A study confidentiality information sheet was included on the first web page of the questionnaire. It indicated that students’ participation was voluntary, and information would be used only for research purposes. A click on “agree” on the consent web page was required from each student before filling out the survey.

### Data collection instrument and procedure

The medical students were asked to complete a structured questionnaire that had been designed and formulated based on the information provided by the Maslach Burnout Inventory – Student Survey (MBI-SS) [7], which calculates a burnout score using 16 items for three categories of burnout symptoms EE, DP, and PA [7]. Additionally, the questionnaire had been modified to include demographic data (age, gender, medical college, and school year).

The questionnaire consisted of two parts. Part 1 identified sociodemographics and personal characteristics of the participants, such as age, gender, academic year, and medical college. Part 2, MBI-SS, was used to determine burnout among the respondents. Schaufeli validated it, showing acceptable reliability among students in Spanish, Portuguese, and Dutch [7]. Among Chinese students, the MBI-SS has been found to have sufficient reliability and factorial validity [8]. The survey contains 16 items that focus on the triad of exhaustion (five items), cynicism (five items), and professional efficacy (six items). Exhaustion refers to the student’s exhaustion induced by academic demands, cynicism refers to the student’s detachment from lessons, and professional effectiveness refers to the student’s academic achievement. All items on the survey were graded on a 7-point Likert scale ranging from 0 (*Never*) to 6 (*Always*). Scores were categorized into high, moderate, or low scores in each of the three components. High scores on cynicism and exhaustion and low scores on professional efficacy indicate high burnout [8]. In this study, Cronbach’s alpha for the MBI-SS was 0.77 for professional efficacy, 0.87 for exhaustion, and 0.75 for cynicism.

### Statistical analysis

An analysis of variance was used to compare groups and test interactions. Components of the MBI-SS subscores for emotional exhaustion, cynicism, and personal efficacy were analyzed for gender, age, school year, and college.

## Results

A total of 336 students were included in this study, with 222 participants from Al-Mulayda College and 114 participants from Unayzah College. The majority of the participants (*n* = 287; 85.4%) were young (18–24 years old). Among the 336 participants, *n* = 190 (56.5%) were female. Of the total participants, *n* = 194 (57%) were in the preclinical years, while *n* = 142 (42.3%) were in the clinical years, including the internship year. See Table 1.

**Table 1:** Demographic characteristics of the participants (*N* = 336)

Characteristics	Categorize	***n*** (%)
**Age**	18–24	287 (85.4%)
	25–34	49 (14.6%)
	35–44	0 (0%)
**Gender**	Male	146 (43.5%)
	Female	190 (56.5%)
**School year**	First	42 (12.5%)
	Second	91 (27.1%)
	Third	61 (18.2%)
	Fourth	68 (20.2%)
	Fifth	56 (16.7%)
	Internship year	18 (5.4%)
**College**	Buraydah	222 (66.1%)
	Unayzah	114 (33.9%)
**Academic year**	Preclinical	194 (57.7)
	Clinical	142 (42.3)

Approximately 8% of medical students have a high EE level, cynicism, and a low level of personal efficacy. However, 18% have a high level of both EE and cynicism. See Tables 2 and 3.

**Table 2:** Comparison of MBI-SS subscales with students’ demographic

		Emotional exhaustion			Cynicism			Personal efficacy		
		≤17 Low	18–25 Mod.	≥26 High	≤12 Low	13–19 Mod.	≥20 High	≥26 Low	19–25 Mod.	≤18 High
**Age**	18–24	87 (30.3%)	121 (42.2%)	79 (27.6%)	93 (32.4%)	103 (35.8%)	91 (31.7%)	100 (34.8%)	101 (35.2%)	86 (30.1)
	25–34	17 (34.7%)	12 (24.5%)	20 (40.8%)	16 (32.7%)	12 (24.5%)	21 (42.9%)	14 (28.6)	14 (28.6)	21 (42.9%)
**Gender**	Male	68 (46.6%)	55 (37.7%)	23 (15.8%)	49 (33.6%)	57 (39.0%)	40 (27.4%)	58 (39.7%)	52 (35.6%)	36 (24.7%)
	Female	36 (18.9%)	78 (41.1%)	76 (40%)	60 (31.6%)	58 (30.5%)	72 (37.9%)	56 (29.5%)	63 (33.2%)	71 (37.4%)
**College**	Buraydah	74 (33.3%)	84 (37.8%)	64 (28.8%)	68 (30.6%)	75 (33.8%)	79 (35.6%)	76 (34.2%)	70 (31.5%)	76 (34.2%)
	Unayzah	30 (26.3%)	49 (43.1%)	35 (30.7%)	41 (36.1%)	40 (35.1%)	33 (30.0%)	38 (33.3%)	45 (39.5%)	31 (27.2%)
**School year**	First	8 (19.0%)	26 (61.9%)	8 (19.0%)	16 (38.1%)	13 (30.9%)	13 (30.9%)	15 (35.7%)	13 (31.1%)	14 (33.3%)
	Second	33 (36.3%)	36 (39.6%)	22 (24.2%)	27 (29.7%)	37 (40.7%)	27 (29.7%)	31 (34.1%)	33 (36.3%)	27 (29.7%)
	Third	17 (27.9%)	28 (45.9%)	16 (26.2%)	20 (32.8%)	23 (37.7%)	18 (29.5%)	20 (32.8%)	23 (37.7%)	18 (29.5%)
	Fourth	20 (29.4%)	19 (27.9%)	29 (42.6%)	20 (29.4%)	26 (38.2%)	22 (32.4%)	17 (25%)	28 (41.2%)	23 (33.8%)
	Fifth	15 (26.8%)	18 (32.1%)	23 (41.1%)	16 (28.6%)	12 (21.4%)	28 (50%)	21 (37.5%)	15 (26.8%)	20 (35.7%)
	Internship	11 (61.1%)	6 (33.3%)	1 (5.6%)	10 (55.6%)	4 (22.2%)	4 (22.2%)	10 (55.6%)	3 (16.7%)	5 (27.8%)
**School year**	**Preclinical**	58 (29.9%)	90 (46.4%)	46 (23.8%)	63 (32.5%)	73 (37.6%)	58 (29.9%)	66 (34.0%)	69 (35.6%)	59 (30.4%)
	**Clinical**	46 (32.4%)	43 (30.3%)	53 (37.3%)	46 (32.4%)	42 (29.6%)	54 (38.0%)	48 (33.8%)	46 (32.4%)	48 (33.8%)

**Table 3:** Total number of participants suffering from a high level of MBI-SS

	*N* (%)
EX and CY (*high level*)	61 (18.2%)
EX, CY, and PE (*high level*)	30 (8.9%)

Table 3 shows that the total number of participants suffering from a high level of MBI-SS was 30 of the 336 students (8.9%).

The percentage of medical students in all domains was divided into approximately 30% for each severity level. Precisely, 29.5% had high EE, 39% had moderate EE, and 31% had low EE. Also, 33% of students had high cynicism, 34% had moderate cynicism, and 32% had low cynicism. Moreover, 33.9% exhibited a high level of personal efficacy, while 34.2% and 31.8% showed a moderate and low personal efficacy level, respectively. Overall, the mean of all medical student participants was in the moderate range of severity for EE (20), CY (16), and PA (21). See Table 4.

**Table 4:** Mean scores and levels of MBI-SS subscales

MBI-SS	*M* (*SD*)	*n*	Percent
Emotional exhaustion			
≤ 17 (*low level*)	20.38 (7.051)	104	31.0%
18–25 (*moderated level*)		133	39.6%
≥ 26 (*high level*)		99	29.5%
Cynicism			
≤ 12 (*low level*)	16.31 (6.998)	109	32.4%
13–19 (*moderated level*)		115	34.2%
≥ 20 (*high level*)		112	33.3%
Personal efficacy			
≥ 26 (*high level*)	21.59 (7.641)	114	33.9%
19–25 (*moderated level*)		115	34.2%
≤ 18 (*low level*)		107	31.8%

Regarding the impact of gender difference on the level of burnout, there was a significant difference observed between males and females in EE (*p* value = 0.000) and personal efficacy (*p* = 0.032), while there was no significant difference in cynicism (*p* value = 0.1). See Tables 5 and 6. Logistic regression is performed as shown in Table 6, which shows that the female gender was a significant predictor of emotional exhaustion (OR = 2.510; 95% Cl = [1.845–3.415]; *p* value 0.000). Similarly, the female gender was a significant predictor of personal efficacy [OR = 1.434; 95% Cl [1.086–1.866]; *p* value 0.010]. According to the comparison between the level of burnout and demographic characteristics, Table 6 shows a significant difference in EE but no significant difference in EE and cynicism regarding participant age. There was no significant difference observed between Al-Mulayda and Unayzah in the level of burnout (see Table 6). The students in their clinical years found to have a higher level of EE was statistically significant (*p* = 0.005) compared to preclinical years. See Table 5.

**Table 5:** Comparison of differences in the MBI-SS subscales according to students’ demographics (using the chi-square test (*χ*^2^) (2-sided))

	Emotional exhaustion *χ*^2^ (***p*** value)	Cynicism *χ*^2^ (***p*** value)	Personal efficacy *χ*^2^ (***p*** value)
**Age**	6.070 (0.048)	3.151 (0.207)	3.206 (0.201)
**Gender**	37.071 (0.000)	4.578 (0.101)	6.892 (0.032)
**College**	1.792 (0.408)	1.694 (0.429)	2.579 (0.275)
**School year**	30.477 (0.001)	15.310 (0.121)	9.007 (0.531)
**Preclinical vs. clinical students**	10.697 (0.005)	3.179 (0.204)	0.538 (0.764)

**Table 6:** Logistic regression analysis for burnout subscales and students’ demographics

Parameter	EE			CY			PE		
	OR	***p*** value	95% CI	OR	***p*** value	95% CI	OR	***p*** value	95% CI
Age	1.159	0.459	[0.784–1.714]	1.181	0.385	[0.811–1.720]	1.341	0.128	[0.919–1.958]
Gender	2.510	0.000	[1.845–3.415]	1.210	0.163	[0.999–1.064]	1.424	0.010	[1.086–1.866]
College	1.159	0.321	[0.866–1.551]	0.833	0.201	[0.630–1.102]	0.911	0.511	[0.689–1.204]
School year (preclinical and clinical)	1.203	0.196	[0.909–1.591]	1.133	0.360	[0.867–1.481]	1.056	0.687	[0.809–1.380]

## Discussion

To our knowledge, this is the first study to assess the prevalence of burnout among two medical colleges, Al-Mulayda and Unayzah medical colleges, in the Qassim region in KSA. Moreover, it was conducted during the critical period, the beginning of the COVID-19 pandemic at which the University has just suspended physical attendance to schools and universities. Burnout prevalence in our study was 8% if the definition of burnout is having a high level of EE and cynicism and a low level of PA, which is roughly in agreement with a recent study conducted in Unayzah Medical College which reported the burnout prevalence to be 5.6% [9]. However, if we limit the definition of burnout to only a high level of EE and cynicism, the percentage of medical students with burnout in our study increases to 18%. This percentage is much lower than a result of a systemic review, which showed the prevalence of burnout among nine studies to be between 45 and 71% [10]. In our study, around 30% of medical students had either high EX or PA or CY. Although this is a high percentage, it is slightly lower than another study that used a similar instrument (MBI-SS) and percentile cut scores [11]. A local study conducted in Saudi Arabia showed a much higher percentage of medical students with high EE and CY (58, 62) compared to our result, but lower regarding PA (60) [12]. A possible reason for low PA compared to other studies might be because this study was conducted during the COVID-19 pandemic, during which time distance learning was implemented. A systemic review showed a summary range of means of MBI HSS of different studies as 22.8 and 35 [13]. This indicates there is a higher mean in our study regarding cynicism and PA, but lower in EE (EE 20, CY 16, PA 21).

Although several studies have reported that females are more likely to experience burnout than males, systemic reviews have shown no significant difference [14]. However, one review showed that female burnout is more likely to be presented with high EE and male burnout is more likely to be presented with high depersonalization [5]. Our study showed that females significantly have higher EE than males, but there was no significant difference between females and males regarding cynicism.

Qassim University has two medical colleges. First, Al-Mulayda Medical College uses problem-based learning (PBL). Second, Unayzah Medical College uses the team-based learning (TBL) style. There was no significant difference between the medical colleges. A study that compared burnout among students in traditional teaching showed no significant difference [15]. Although further research is needed, this possibly emphasizes the importance of factors other than teaching style.

There are several strengths and limitations to our study. Our study included two medical colleges, both genders, and all medical college years, including interns. Moreover, it used a validated questionnaire. The study’s timing was unique as it was conducted during the beginning of distance learning due to COVID-19. The study’s limitations include a low response rate, it was cross-sectional, and there was no comparison with other health colleges (e.g., dentistry college).

### Recommendations

Our study shows that burnout is prevalent among medical students. No change in medical education style is needed to reduce burnout, based on our study results. However, further studies to confirm such findings are required.

Searching for other factors that might contribute to burnout is required in future research. Education in Qassim Medical College is in English. Students have different degrees of mastering the English language. We speculate that students with difficulty using the English language are at higher risk of developing burnout. Other factors suggested to be studied include the association between burnout and social factors, and psychological and biological illnesses among medical students.

Implementing strategies that might decrease burnout are recommended, including problem-solving, constructive reinterpretation, and emotional expression. Additional recommendations include establishing student-led services to encourage senior students’ mentorship of junior students and encouraging students to get regular physical exercise and enough sleep to improve their wellbeing [15].

## Conclusion

Among study participants, significant levels of burnout were observed. Several interventions are required to decrease medical students’ burnout, as it can accumulate over the years and contribute to negative impacts on student mental health and social life, including employment. Longitudinal research is essential to investigate the burnout trend of medical students from entry to graduation.

## Data Availability

The datasets used and/or analyzed during the current study are available from the corresponding author on reasonable request.
